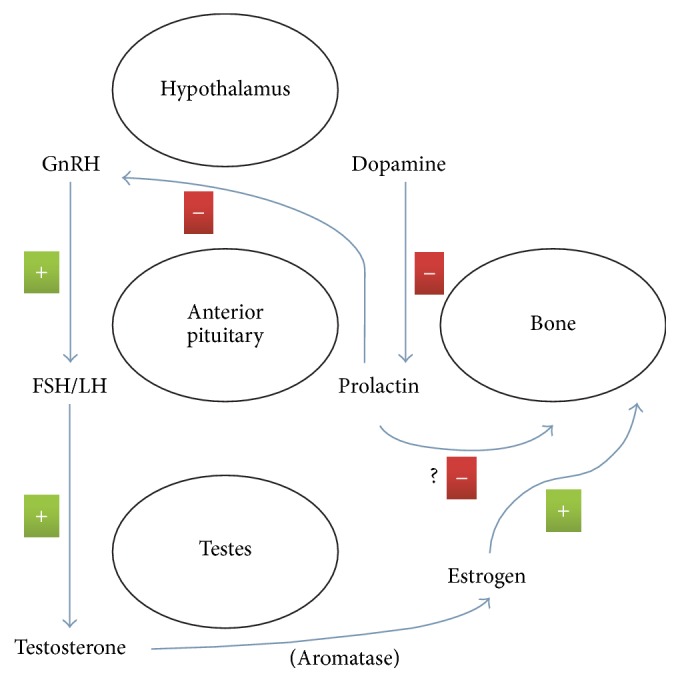# Prolactinoma: A *Massive* Effect on Bone Mineral Density in a Young Patient

**DOI:** 10.1155/2016/6312621

**Published:** 2016-06-30

**Authors:** Scott Sperling, Harikrashna Bhatt

**Affiliations:** Warren Alpert Medical School, Brown University, 900 Warren Avenue, Suite 300, East Providence, RI 02914, USA

## Abstract

This case highlights a prolactinoma in a young male, and its impact on bone health. Osteoporosis has been noted to be an issue in postmenopausal women with prolactinomas. This case shows a similar impact on bone health in a young male resulting in low bone mineral density for age based on *Z*-score. This case report highlights the possible mechanisms for the bone loss in the setting of prolactinoma and the need for assessing bone health in such patients. Furthermore it highlights the need for a thorough evaluation in such patients.

## 1. Introduction

Osteoporosis is a major health concern, affecting millions of Americans, and is a result of low bone mineral density (BMD) with microarchitectural disruption leading to increased risk of fracture [1]. Many of our current guidelines focus on postmenopausal women, but osteoporosis is becoming an increasingly recognized health problem in men. Prior studies have suggested that all types of major osteoporotic fractures are associated with increased mortality in both sexes, but more so in men [2, 3].

Measurement of BMD by DEXA can be used to diagnose postmenopausal women and men over 50 with osteoporosis based on a calculated *T* score, but premenopausal women and men under 50 can only be characterized as “below the expected range for age” in regard to BMD based on the reported *Z*-score [4], which compares a person's bone density to an average individual of the same age and sex. A *Z*-score below 2.0 at any age may indicate a secondary cause of low BMD and these include primary hyperparathyroidism, hyperthyroidism, prolonged glucocorticoid use, malabsorption, primary or secondary hypogonadism (medication effect, opioid use, and hyperprolactinemia), and lifestyle decisions (smoking and excessive alcohol use) [5].

## 2. Case Presentation

**Figure 1:**
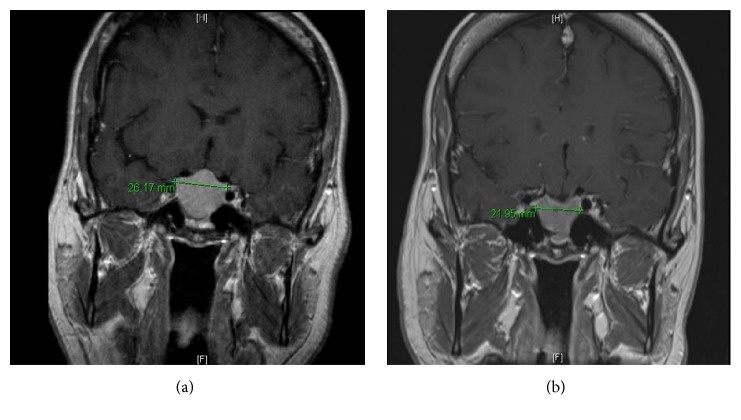


Our patient is a 37-year-old gentleman without significant past medical history who initially presented to his primary care physician with complaints of lumbago and diffuse arthralgias. An X-ray of his L spine was concerning for decreased bone mass and a DEXA scan was notable for a *Z*-score of −3.6. Physical examination revealed intact neurologic function. Secondary workup was significant for normal renal and liver function. Celiac disease, multiple myeloma, and glucocorticoid excess were excluded with tissue transglutaminase and anti-endomysial antibodies, SPEP/UPEP, and 24-hour urine cortisol and dexamethasone suppression test, respectively. Additional endocrinologic evaluation revealed normal thyroid function. His calcium and phosphorus were normal, while he was noted to be vitamin D deficient (25-OH-Vitamin D level was 20.7 ng/mL). His prolactin was elevated to 974 ng/mL (normal 2–17), with low FSH and low-normal LH and testosterone. The elevated prolactin prompted further investigation with an MRI of the brain that illustrated a pituitary macroadenoma measuring up to 3.2 cm with extension into the sphenoid sinuses and mild superior mass effect upon the optic nerves (Figure 1(a)). Formal ophthalmologic examination revealed normal visual fields. In consultation with endocrinology, the patient was started on cabergoline, a dopamine agonist, 0.25 mg twice weekly along with calcium and vitamin D supplementation. Repeat prolactin levels two months after initiation of therapy were 568 and at ten months they continued to trend down to 290, and 25-OH-Vitamin D level after supplementation was 30 ng/mL. A follow-up MRI at 6 months revealed significant interval decrease in size with a superior border to the lesion which no longer impinged upon the optic chiasm (Figure 1(b)). Furthermore with improvement of prolactin, the patient's bone density also showed improvement. At this point, the patient has elected to continue monitoring his bone density and has decided not to pursue pharmacological therapy for the low bone density. He has continued dopamine agonist therapy for the prolactinoma and the patient has responded to this therapy.

## 3. Discussion

Studies on hyperprolactinemia resulting in bone loss have generally focused on postmenopausal women, but here we present a case of BMD below the expected range in a 37-year-old gentleman. While women with prolactinomas tend to present with microadenomas due to earlier recognition of the endocrine effects, including galactorrhea and amenorrhea, men tend to present with macroadenomas (10 mm or greater) resulting in headaches, visual symptoms, and hypogonadism, with the latter resulting in decreased libido and impotence [6]. There are limited case reports and series in the literature that focus on the resultant bone loss and osteoporosis in men [7–9], and this case is unique because our patient came to medical attention with markedly decreased BMD, as opposed to the other abovementioned symptoms.

Hypogonadism is thought to be the main mechanism in which these individuals develop low BMD, which is a result of abnormalities in the normal pulsatile secretion of GnRH due to elevated prolactin levels [1]. This hypothesis is supported by a study that shows that when testosterone levels are restored to within normal limits, irrespective of prolactin levels, there is improvement in BMD [10]. Prolactin may have a direct effect on bones; however the exact mechanism is not well established. There is evidence that prolactin may have a direct effect on osteoblasts. An in vitro study showed the expression of prolactin receptors on osteoblasts, and prolactin treated osteoblasts showed decreased proliferation with an overall increased rate of apoptosis. There was also decreased calcium content in these cells, thus suggesting decreased mineralization [11]. A study of vertebral fractures in men with prolactinoma also suggests a direct effect of prolactin on BMD as men with increased prolactin levels showed increased radiographic evidence of vertebral fractures despite having similar testosterone levels to men without fractures [12]. Also of note, in this study there was a high rate of vertebral fractures when elevated prolactin was noted in hypogonadal subjects; however it was also noted in subjects who were eugonadal. This suggests that prolactin may have a sex hormone-independent effect on skeletal integrity [12]. There was a significantly increased rate of osteopenia, osteoporosis, and ultimately vertebral fractures in a study comparing women with prolactinomas to those with normal prolactin levels. Those with prolactinomas and a fracture had a higher serum prolactin level and lower BMD than women with a prolactinoma and no radiographic evidence of vertebral fracture [13]. This patient indeed had improvement in bone density after lowering of the prolactin, and this point underscores the indirect effect of prolactin on bone strength via testosterone. Furthermore this case lends evidence to the possible direct negative effect of prolactin on osteoblasts.

Once a diagnosis of hyperprolactinemia is established there must be additional workup to investigate the underlying cause before attributing it to a prolactinoma, as this will ultimately lead to the appropriate treatment. A careful medication review must be performed as those with dopamine-antagonist activity, including antipsychotics, methyldopa, and opioids, can elevate prolactin levels. This is a result of the normal tonic* inhibition* that dopamine plays in the secretion of prolactin by the anterior pituitary and the rationale behind using dopamine-agonists (cabergoline, bromocriptine) in the treatment of prolactinomas. Another consequence of this is the elevation of prolactin in the setting of pituitary stalk lesions or mass effect since the dopamine originates from the hypothalamus [1]. Other medical diseases including CKD and liver disease must also be ruled out as prolactin is cleared by the kidney (25%) and liver (75%) with organ dysfunction resulting in buildup of the hormone. Hypothyroidism must also be evaluated for, as TRH stimulates prolactin secretion [1].

This case highlights how hypogonadism in a young male warrants a proper and thorough evaluation, and, regardless of the underlying cause of the hypogonadism, bone mineral density may need to be evaluated in such patients. Further studies will be needed to investigate long term effects of prolactinomas in men. Additionally, as there is evidence of a direct effect of prolactin on osteoblasts in vitro, follow-up studies looking at the effects in vivo could help lead to development of therapeutics. Possible mechanisms for the bone loss in the setting of prolactinoma likely involve several pathways (Figure 2).

## Figures and Tables

**Figure 2:**